# Reduced inflammation and altered innate response in neonates during paramyxoviral infection

**DOI:** 10.1186/1743-422X-8-549

**Published:** 2011-12-20

**Authors:** Somashubhra Bhattacharya, Brandon T Beal, Ann M Janowski, Laurie P Shornick

**Affiliations:** 1 Department of Biology, Saint Louis University, 3507 Laclede Avenue, Saint Louis, MO 63103, USA; 2 Department of Molecular Microbiology & Immunology, Saint Louis University, Saint Louis, MO, USA

**Keywords:** Viral, Neonatal, Lung, Innate

## Abstract

**Background:**

Human infants are frequently hospitalized due to infection with the paramyxovirus respiratory syncytial virus (RSV). However, very little is known about the neonatal response to paramyxoviral infection. Here, a neonatal model of paramyxoviral infection is developed using the mouse pathogen Sendai virus (SeV).

**Results:**

Adult mice infected with SeV developed a predominantly neutrophilic inflammatory cell influx and a concomitant reduction in lung function, as determined by oxygen saturation. In contrast, neonates with SeV had significantly reduced inflammation and normal lung function. Surprisingly, infected neonates had similar viral loads as adult mice. A reduced neutrophil influx in the neonates may be due in part to reduced expression of both CXCL2 and intracellular adhesion molecule-1 (ICAM-1). Expression of IFN-γ and TNF-α increased in a dose-dependent manner in adult lungs, but neonates did not increase expression of either of these cytokines, even at the highest doses. Importantly, the expression of the RIG-I-like receptors (RLRs) was delayed in the neonatal mice, which might have contributed to their reduced inflammation and differential cytokine expression.

**Conclusions:**

Neonatal mice developed similar SeV titers and cleared the virus with similar efficiency despite developing a dramatically lower degree of pulmonary inflammation compared to adults. This suggests that inflammation in the lung may not be required to control viral replication. Future studies will be needed to determine any effect the reduced inflammation may have on the development of a protective memory response in neonates.

## Background

Acute respiratory infection is the leading cause of mortality in young children, accounting for 20% of childhood deaths worldwide [1]. The most common viral respiratory pathogen in infants and children is the paramyxovirus respiratory syncytial virus (RSV). RSV induces a clinically significant bronchiolitis in human infants, which may result in hospitalization [2]. Hospitalization rates are highest for infants between 3 and 6 months of age, whereas infants less than 1 month of age have the lowest rate of RSV hospitalization [3]. This may be due to reduced exposure in this age range or due to decreased severity of respiratory disease as some studies suggest that the youngest infants infected with RSV may be asymptomatic [4,5]. The increased susceptibility to respiratory viral infection in infants is attributed to deficiencies in both the innate and adaptive immune responses (reviewed in ([6,7]) respectively).

Despite the fact that infants are the most at risk population for serious respiratory viral infection, the neonatal immune response to RSV is poorly understood. During the 1960s, an attempt to develop a vaccine for RSV failed when the infants that received the vaccine developed enhanced respiratory disease upon subsequent infection [8]. Since that time, there have been many studies of RSV infection in adult animals but relatively few studies examining RSV and paramyxoviral infection in neonates (reviewed in [9]). Here we describe the immune response of neonatal mice to the paramyxovirus Sendai virus (SeV). When compared to adult animals, newborn mice developed similar SeV titers and cleared the virus with similar efficiency despite developing a dramatically lower degree of pulmonary inflammation. These observations, together with recent studies by others using RSV and pneumonia virus of mice (PVM), suggest that inflammation can be decoupled from viral clearance and may necessitate a reassessment of the paradigm of immune responses to RSV infection in human infants [10,11].

## Results

**Figure 1:**
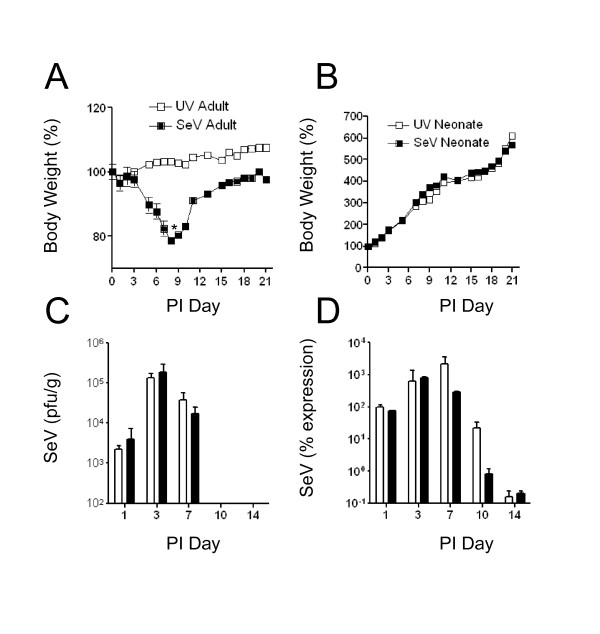
**Adult and neonatal mice displayed differential body weight patterns during viral infection, but had a similar viral load in the lung**. **a**) 6-8 week old C57BL/6 adult mice (n = 21) were inoculated intranasally with 500 pfu/g body weight SeV in sterile PBS (black squares). The control group was inoculated with an equivalent amount of UV inactivated SeV (white squares). **b**) Neonatal C57BL/6 mice (n = 31) were infected on postnatal day 2 with 500 pfu/g body weight SeV (black squares) or with UV-SeV (white squares). **c**) Viral plaque assay of whole lung homogenates was performed, and pfu/ml was normalized to weight of the lung tissue (g). **d**) Real-time PCR for SeV was performed on whole lung RNA. SeV expression was normalized to GAPDH and percent expression was calculated by the 2(-Delta Delta C(T)) method. Adults (white bars) and neonates (black bars). All values represent means ± SD; SD of adult SeV group was ≤ 1.24. SD of neonatal groups was ≤ 1.10. *s = 0.007, **p = 0.030

Six eight-week old adult C57BL/6 mice and postnatal day 2 neonatal C57BL/6 mice were infected with 500 pfu/g body weight SeV via intranasal inoculation. The virus was administered in a volume of 30 μl for the adult mice, and 6 μl for the neonatal mice. Control groups were inoculated with an equivalent amount of SeV that was inactivated by exposure to ultraviolet light. Infection of adult mice with SeV resulted in body weight loss that peaked at day 9 post-infection. The adult mice then began to regain weight on post-infection day 12. This weight loss was not observed in adult mice inoculated with UV-inactivated SeV (Figure 1a). In contrast to the adults, the neonatal mice were in a period of rapid growth. At the 500 pfu/g body weight dose of SeV, there was no difference in body weight in the infected neonates compared to the UV-inactivated control group (Figure 1b).

Infectious virus in lung homogenates was quantified by viral plaque assay (Figure 1c). There was an equivalent level of virus per gram of lung tissue in the adults and neonates at 24 hours post-infection, which demonstrated that the mice were infected with equivalent doses of virus per gram of body weight. Viral replication in both the adults and neonates peaked on day 3, and levels remained equivalent on day 7. In both adults and neonates, live infectious virus was not detectable by days 10 and 14 post-infection, suggesting that there was no difference in viral clearance between the two groups. Real-time PCR for SeV was performed and normalized to GAPDH expression in the lung. This method also demonstrated equivalent levels and clearance of SeV RNA in both adult and neonatal lungs at all time points through day 14 (Figure 1d).

**Figure 2:**
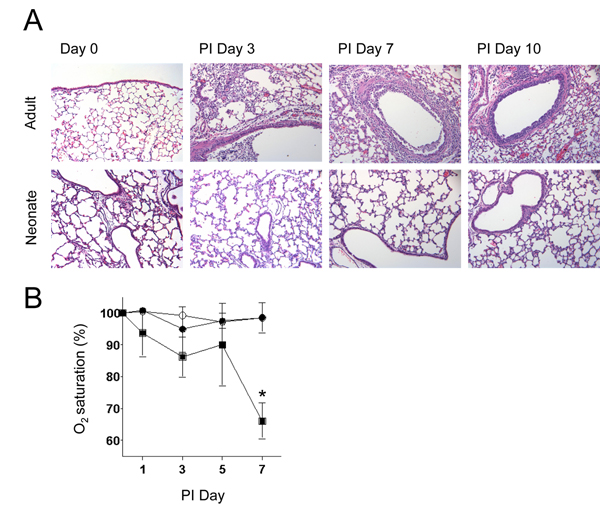
**Neonatal lungs had normal lung function and an absence of inflammatory cell influx**. Adult and neonatal C57BL/6 mice were infected as described in Figure 1. **a**) Lung sections of adult and neonatal mice at untreated day 0 and post-infection days 3, 7, and 10 were stained with hematoxylin and eosin (magnification 200X). **b**) Oxygen saturation, as a measure of lung function, was analyzed using a mouse pulse oximeter. Black squares denote adults inoculated with SeV (n = 6), white circles are UV-SeV inoculated neonates (n = 3), and black circles are SeV inoculated neonates (n = 6). All values represent the mean ± SD; *p = 0.029

Despite having equivalent levels of virus, the neonates had significantly less inflammation in the lung during SeV infection at all times post-infection (Figure 2a). In order to determine if the level of inflammation correlated with lung function, pulse oximetry was used to measure oxygen saturation. Reduced oxygen saturation was observed in the adult mice at the peak of infection, but there was no loss of oxygen saturation in the neonates. Thus, normal levels of oxygen saturation correlated with the absence of inflammation in the lungs of infected neonates (Figure 2b).

**Figure 3:**
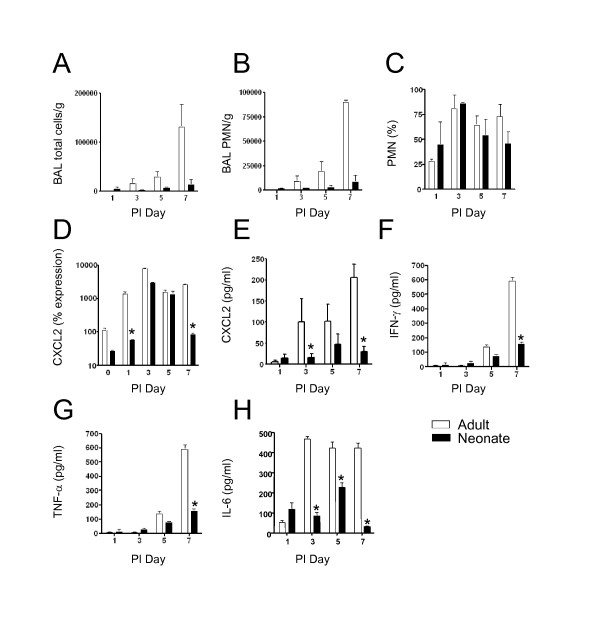
**Neutrophil influx and CXCL2 expression were lower in neonatal lungs**. Adult and neonatal C57BL/6 mice were inoculated with SeV 500 pfu/g body weight. **a**) On the indicated days, BAL was performed and total BAL fluid cells were counted. **b**) To enumerate the neutrophils, BAL fluid cells were subjected to Wright Giemsa staining and differential cell counting. **c**) Percentage of neturophils of total BAL cells. **d**) Whole lung RNA was analyzed by real-time PCR for CXCL2. *p < 0.05. Whole lung protein homogenates were analyzed for **e**) CXCL2, **f**) IFN-γ, **g**) TNF-α, and **h**) IL-6 protein by ELISA. *p < 0.05. White bars denote adults, and black bars denote neonates. N = 4 at each time point. All values represent the mean ± SD

Total cell counts of bronchoalveolar lavage (BAL) fluid showed that there was an increase of cells migrating into the lungs of adult mice, but a minimal increase of cells in the neonatal lungs during viral infection (Figure 3a). Differential cell counts (adjusted for total body weight) showed a predominantly neutrophilic influx in the adult lungs that peaked at day 7. The total number of neutrophils was lower in the neonatal lungs, although the percentage of neutrophils in the BAL of adults and neonates was similar (Figure 3b and 3c). Total macrophage and lymphocyte numbers were also increased in the adult lungs compared to the neonates, but these were not statistically significant (data not shown). The neutrophil chemoattractant CXCL-2 (Macrophage Inflammatory Protein-2; MIP-2) was significantly greater in the adult lungs. CXCL-2 mRNA increased after infection in both the adults and neonates, and this increase peaked in both groups on post-infection day 3 (Figure 3d). CXCL2 protein levels peaked in the neonate on day 5 post-infection, but were decreased on day 7. CXCL2 protein levels in the adult were significantly higher than neonatal levels on days 3 and 7 (Figure 3e). In addition, differential cytokine expression was observed between the adults and neonates during the course of infection. Both IFN-γ and TNF-α increased during the course of infection in adult lungs. However, the levels of these cytokines in neonatal lungs were significantly lower in neonates on post-infection day 7 (Figure 3f and 3g). IL-6 levels were upregulated by day 3 in adults and remained elevated through day 7. However, the levels were significantly lower in neonates at these times (Figure 3h). Thus, the increased CXCL2 levels as well as the increased IFN-γ, TNF-α and IL-6 corresponded with the higher levels of inflammation in the adult lungs.

**Figure 4:**
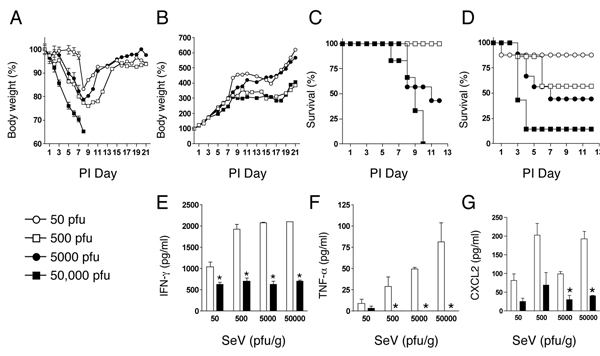
**Neonates had reduced expression of IFN-γ, TNF-γ and CXCL2 even at higher doses of virus**. Adult (n = 3 for each dose) and neonatal C57BL/6 mice (n = 6 for each dose) were infected with 50, 500, 5000, or 50,000 pfu SeV/g body weight. **a**) Body weight loss in adults **b**) Body weights in neonates. White circles 50 pfu/g body weight, black circles 500 pfu/g, white squares 5,000 pfu/g, and black squares 50,000 pfu/g. **c**) Adult survival graph: white circles 50 pfu/g (n = 6), black circles 500 pfu/g (n = 6), white squares 5,000 pfu/g (n = 6) black squares 50,000 pfu/g (n = 6). **d**) Neonatal survival graph: white circles 50 pfu/g (n = 8), black circles 500 pfu/g (n = 9), white squares 5,000 pfu/g (n = 8) black squares 50,000 pfu/g (n = 7). **e**) Post-infection day 7 IFN-γ protein expression was determined by ELISA. *p < 0.05. **f**) Post-infection day 7 TNF-α protein expression was determined by ELISA. TNF-α proteins levels were below the level of detection in the neonatal samples at inoculation doses of 500, 5000, and 50,000 pfu/g body weight (*none detected). **g**) Post-infection day 7 CXCL2 protein expression was determined by ELISA. Adults (white bars); Neonates (black bars). Data are means ± SD.

**Figure 5:**
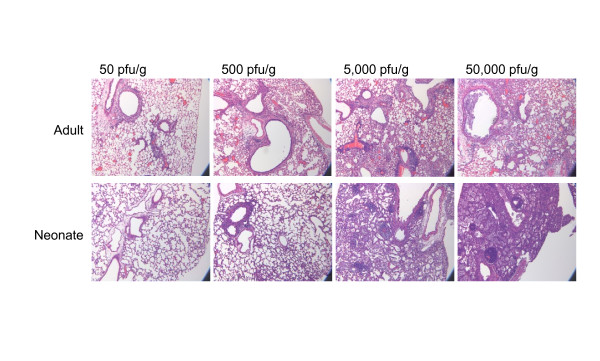
**Neonatal lungs demonstrated inflammation at higher doses of SeV**. Adult and neonatal mice were infected as described in Figure 4. Post-infection day 7 lungs were stained with hematoxylin and eosin (magnification 100X).

In order to compare the adult and neonatal responses in a more comprehensive manner, a dose response of viral infection was performed. Adult and neonatal mice were infected with SeV at the doses of 50, 500, 5000 or 50,000 pfu/g body weight. The adult mice showed the expected dose dependent decreases in body weight during the first week of infection, and the adults in the 50,000 pfu/g and 5,000 pfu/g groups lost approximately 30% of their body weight by day 7 (Figure 4a). There was 100% survival of the adults in the 50 and 500 pfu/g doses, but only 43% at the 5,000 pfu/g dose. None of the adults survived the highest dose of 50,000 pfu/g beyond post-infection day 10. In contrast, the neonatal mice continued to grow during the first week post-infection. There was no difference in the growth curve until day 11 when there was a significant slowing of growth of the neonates that received 5,000 pfu/g and 50,000 pfu/g doses of SeV (Figure 4b). In addition, reduced survival was observed in the neonatal mice in a dose dependent fashion (Figure 4d), and there was inflammation in the lungs at the highest doses (Figure 5). Notably, even at these higher doses, the inflammation in the neonates was due to a differential cytokine expression pattern as compared to adult lungs. On day 7 post-infection, protein levels of IFN-γ, TNF-α, and CXCL2 in the adult lung increased with higher doses of SeV. However, the levels of IFN-γ were significantly lower in the neonatal lungs (Figure 4e), and TNF-α was at or below the level of detection in the neonates at all doses of virus (Figure 4f). In addition, significantly higher CXCL2 protein expression was observed in the adults (Figure 4g). Thus, the inflammation that was present in the neonates at the higher doses involved different cytokine signals compared to the adult mice.

**Figure 6:**
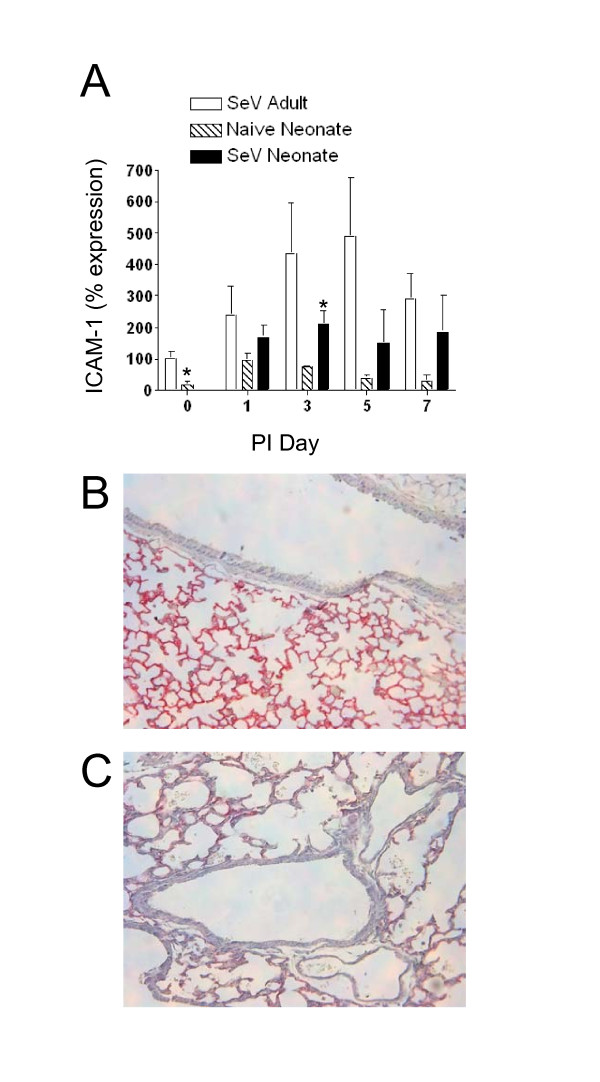
**ICAM-1 mRNA and protein levels were lower in neonatal lungs**. **a**) Adult and neonatal C57BL/6 mice were infected as described in Figure 1. Whole lung RNA from adults (white bars), neonates (black bars), and naïve age-matched neonatal controls (striped bars) was analyzed by qPCR for ICAM-1 mRNA levels. Data are means ± SD. *p < 0.039. ICAM-1 immunostaining. Sections of naïve day 0 untreated adult (**b**) and untreated neonatal lungs (**c**) were stained with an anti-ICAM-1 antibody. Primary anti-ICAM-1 antibody was detected using an alkaline phosphatase red substrate and the sections were counterstained with hematoxylin (magnification 200X)

The reduced inflammatory influx into the neonatal lungs during viral infection led us to hypothesize that this could be due, in part, to decreased expression of adhesion molecules. Real-time PCR for ICAM-1 mRNA expression showed significantly reduced levels in untreated neonates compared to untreated adults (Figure 6a). After viral infection, neonatal ICAM-1 mRNA increased slightly, but was significantly lower than the adult levels on post-infection days 3 and 5. Baseline ICAM-1 protein expression was visualized by immunostaining. There was constitutive level of ICAM-1 protein present in the parenchyma of untreated adult lungs (Figure 6b). In contrast, ICAM-1 protein is barely detectable in the parenchyma of untreated neonatal lungs at day 0 (Figure 6c). The pattern of adult and neonatal ICAM-1 protein expression during the course of viral infection was similar to ICAM-1 mRNA expression (data not shown).

**Figure 7:**
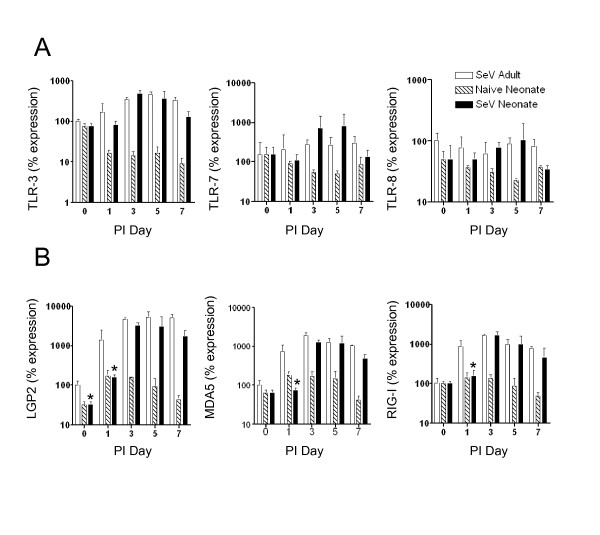
**TLR mRNA expression was similar in adult and neonatal lungs, but RLR mRNA expression was delayed in neonatal lungs**. Adult and neonatal C57BL/6 mice were infected as described in Figure 1. Whole lung RNA isolated from adults (white bars), neonates (black bars) and naïve age-matched neonatal controls (striped bars) was analyzed by real-time PCR for TLR expression **a**) TLR-3, TLR-7, and TLR-8, and RLR expression **b**) LGP2, MDA-5, and RIG-I. All values represent the mean ± SD; *, significant difference from corresponding adult sample.

**Figure 8:**
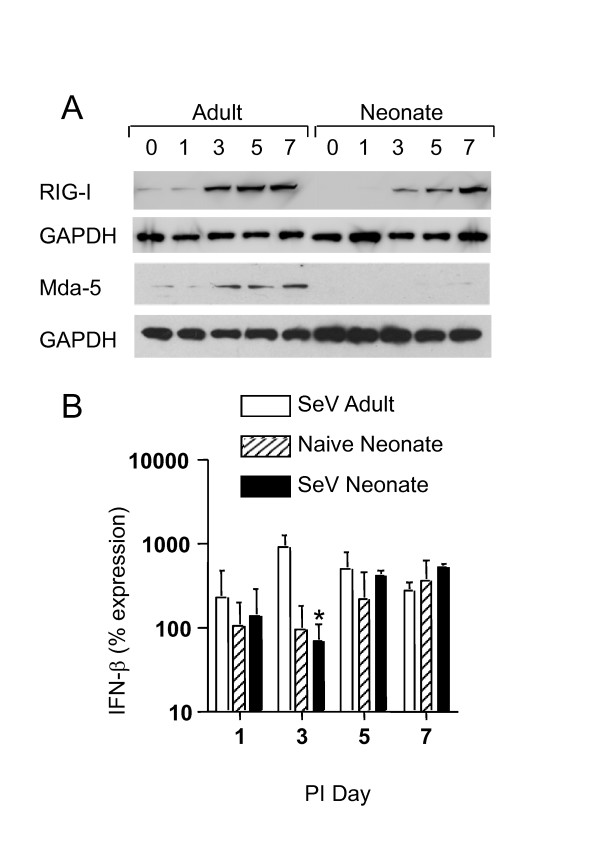
**RLR protein expression was delayed in neonatal lungs, and type I interferon was reduced on day 3 post-infection**. Adult (white bars) and neonatal mice (black bars) were infected as described in Figure 1. **a**) On the indicated days, lungs were homogenized and equivalent amounts of protein were analyzed by SDS-PAGE and western blot analysis. **b**) Whole lung RNA, from adults (white bars), neonates (black bars) and naïve age-matched neonatal controls (striped bars), was analyzed for IFN-β expression by qPCR. All values represent the mean ± SD; *, significant difference from corresponding adult sample.

Because there was a difference in inflammation and chemokine expression as early as 24 hours after infection, we hypothesized that the initial innate response to SeV may be altered in neonatal lungs. Therefore, we examined the expression of the pattern recognition receptors for SeV, which included the Toll-like receptors (TLR3, TLR7 and TLR8) and the RIG-I-like receptors (Lgp-2, Mda-5, and RIG-I). Real-time PCR demonstrated that there was no significant difference in mRNA expression between adults and neonates for TLR3, TLR7, and TLR8 at baseline. After SeV infection, TLR levels in adults and neonates increase in a similar fashion (Figure 7a). In contrast, analysis of the RLRs showed delayed mRNA expression in the neonates for Lgp2, Mda5, and RIG-I (Figure 7b). RLR expression in naïve age-matched neonatal controls did not change during the course of infection, suggesting that the increases in RLR expression were due to viral infection and not merely related to developmental changes. Western blot analysis demonstrated the presence of low levels of RIG-I protein in uninfected adult lungs, but RIG-I protein was not detectable in uninfected neonatal lungs. RIG-I protein increased in the adults on days 3, 5 and 7. RIG-I protein levels also increased in neonatal lungs but this increase was delayed (Figure 8a). In addition, a pattern of delayed expression in neonatal lungs was observed for Mda-5 protein (Figure 8a). No signal was detected in western blot analysis of lung homogenates from naïve age-matched neonatal controls (data not shown). Because the RLRs activate type I interferons, we also examined IFN-β mRNA expression. On post-infection day 3 there was significantly reduced IFN-β mRNA expression, but there was no difference at any of the other time points (Figure 8b).

## Discussion

In this study we developed a neonatal model of respiratory viral infection using the mouse pathogen Parainfluenza 1 virus strain Sendai/52 (Sendai virus; SeV), which is closely related to RSV. SeV and RSV are both enveloped RNA viruses and they share a highly conserved genetic organization [12]. RSV infection in mice does not resemble human RSV bronchiolitis for several reasons (reviewed in [9]). First, RSV infects the airway epithelial cells of humans, but it infects the type I alveolar pneumocyte in the mouse resulting in pneumonia rather than bronchiolitis [13,14]. Secondly, RSV does not replicate well in mice. Inhibition of the interferon (IFN) antiviral response is important for paramyxoviruses to establish replication in the host cell. RSV contains two non-structural proteins NS1 and NS2, which antagonize interferon action by inhibiting STAT1 and STAT2. The RSV NS1 and NS2 molecules are species specific; thus, they can inhibit human STAT1 and STAT2, but they do not inhibit the murine molecules [15]. Finally, in contrast to human RSV infection, RSV infection in mice elicits eosinophilic inflammation and a Th2 cytokine response [16]. In contrast to murine RSV infections, our previous studies have shown that SeV infection of adult mice results in a neutrophilic bronchiolitis and immune response pattern that closely resembles RSV infection in humans [17]. This data suggests that SeV may be a useful model of paramyxoviral infection.

We demonstrated that the neonatal innate immune response to SeV was distinct from the adult response. In adult mice, body weight loss correlated with peak levels of virus in the lung. In contrast, the body weight and viral titer did not correlate in the neonates. Similar patterns of body weight loss in adults and a later slowing of growth in neonatal mice have been observed after infection with RSV, Pneumonia virus of mice (PVM), and the PR8 strain of influenza [10,11,18]. It is possible that the differences of body weight patterns between adults and neonates are reflective of distinct cytokine expression. Some cytokines, including TNF-α, interleukin-1 (IL-1), IFN-γ, and IL-6 have been implicated in cachexia [19]. In our study, the adult mice have significantly increased amounts of IFN-γ and TNF-α, which correlated with their weight loss. However, at higher doses neonates had a slowing of growth (or failure to thrive) after day 9 and this did not correlate with TNF-α expression. In addition, the use of Enbrel to block TNF-α during SeV infection in adult Stat1^-/- ^mice did not affect body weight loss, suggesting that additional cytokines may be responsible for body weight loss during paramyxoviral infection [17].

Importantly, the neonates and adults had comparable viral titers in the lungs, but the neonates exhibited significantly less inflammation. These findings were also consistent with observations made during infection of murine neonates with the paramyxoviruses RSV and PVM. However, neonatal mice infected with the orthomyxovirus influenza H1N1 (Strain A/PR/8/34) had a delayed clearance of virus and increased numbers of inflammatory cells in the interstitium [18]. Therefore, these results of equivalent viral titers and reduced inflammation in the neonates may be unique to paramyxoviral infection.

SeV infection of the airway epithelium causes TNF-α production, which results in the secretion of CXCL2 [17]. In this study, a dose response of SeV caused a dose-dependent increase in TNF-α in the adult mice, but not in the neonatal mice. As expected, the adult mice also produced increased levels CXCL2, which recruited neutrophils into the lungs. In contrast, the neonates did not exhibit an increase in either TNF-α or CXCL2 levels, and there was little to no recruitment of PMN to the lungs. Both TNF-α and CXCL2 are known to increase numbers of circulating neutrophils by releasing them from the bone marrow reserve [20,21]. In addition, neonates have quantitative defects of neutrophil storage pools as well as the capacity to generate neutrophils [22]. Thus, the reduced numbers of PMN in the neonatal lungs may be due to both reduced mobilization from the storage pool and reduced chemokine levels in the lung. Reduced TNF-α and chemokine levels have also been observed in neonates infected with RSV and PVM suggesting that this pattern is consistent in the neonatal response to paramyxoviral infection [10,11].

Even with increased doses of SeV, there was differential cytokine expression in the neonates. The neonates had significantly reduced levels of IFN-γ even at 100-fold higher inoculums of virus. This reduction in IFN-γ did not affect viral clearance in the neonates, which was not unexpected because weight loss, mortality, histology, and cytotoxic CD8+ T lymphocytes numbers were all normal in SeV infected IFN-γ deficient mice, suggesting that IFN-γ is not necessary for paramyxoviral clearance [23,24]. IFN-γ upregulates ICAM-1 expression on airway epithelial cells both *in vitro *and *in vivo *[25]. *In vitro *studies of airway epithelial cells have shown that ICAM-1 expression is increased following paramyxoviral infection and necessary for leukocyte adherence and extravasation [26]. Our observation of reduced ICAM-1 expression may be due to the reduced expression of IFN-γ in neonates. Consequently, the reduced expression of ICAM-1 may contribute to reduced inflammation in neonatal lungs. Previous studies demonstrated that reduced inflammation in adult ICAM-1 knockout mice protected them from weight loss, but did not impair viral clearance, suggesting that inflammation is not necessary for viral clearance [24].

When paramyxoviruses first infect the airway epithelial cells they are detected by pattern recognition receptors (PRR). The PRRs involved in detecting paramyxoviral infection are the Toll-like receptors (the TLRs including TLR-3, TLR-4, TLR-7, and TLR-8) and the retinoic acid-inducible gene 1 (RIG-I)-like receptors (RLRs including RIG-I, Mda-5 and Lgp2). TLR-4 is expressed on the plasma membrane of epithelial cells and it recognizes the fusion (F) protein of hRSV [27]. TLR-3, TLR-7 and TLR-8 are expressed intracellularly on endosomes and they recognize dsRNA and ssRNA, respectively [28-30]. RIG-I and MDA5 are cytoplasmic pattern recognition receptors that recognize viral 5' triphosphate RNA [31,32]. They both contain a DEXD/H helicase domain and 2 caspase-recruitment domains (CARD). Lgp2 (laboratory of genetics and physiology 2) is a helicase that is similar to RIG-I but it lacks the CARD domain and may act as a regulator of RIG-I signaling [33,34]. It has been shown that RIG-I is essential for an appropriate immune response to both the paramyxoviruses RSV and Sendai virus (SeV). In vitro, RIG-I was necessary for type I IFN production in response to SeV infection [34], and RIG-I knockdown during RSV infection of airway epithelial cells inhibited both NFkB and interferon regulatory factor (IRF) signaling [35].

Neonatal TLR responses of monocytes, conventional dendritic cells, and plasmacytoid dendritic cells are distinct from the corresponding adult response to TLR stimulation [36,37]. However, very little is known about PRR expression and activation in neonatal lung during respiratory viral infection. In the present studies, murine neonates had normal expression of the TLRs in the lung at baseline and during paramyxoviral infection; however, all of the RLRs (Lgp2, Mda-5 and RIG-I) had significantly delayed expression during infection. One study of human infants examined PRR mRNA expression in nasal washings of infants hospitalized with respiratory viral bronchiolitis. There was no correlation of PRR expression level and clinical severity score, but there was a positive correlation between RSV viral load and expression of RIG-I [38]. Our results show that RLR expression increases in neonates during the course of infection, so it is possible that these human samples were taken when the infection was well established and RIG-I was already upregulated. Thus, baseline expression of PRRs in human infants is still unknown.

## Conclusions

In summary, our results established a model of paramyxoviral infection that will aid in defining the unique mechanisms of the neonatal immune response. Our data showed that neonates have reduced inflammation in the lungs compared to adults during SeV infection even though both adult and neonatal lungs had a similar viral load and clearance. The inflammation in the adult mice was associated with higher levels of IFN-γ and TNF-α compared to the neonates. This suggests that the cytokine milieu in the neonatal lungs is quite different from the adults. In addition, the neonates have delayed expression of the PRRs (RIG-I, MDa-5 and Lgp2), which are essential for initiating the innate immune response.

Thus, our study with SeV, together with neonatal studies of the related paramyxoviruses, RSV and PVM, all showed reduced inflammatory cell influx in neonates compared to the adults. In all three of these models, neonates were able to control viral replication even with little to no inflammation in the lungs. It is suggested that a strong inflammatory response is important for inhibiting viral replication, the maturation of antigen presenting cells, and the development of a vigorous T cell response. Inflammatory cytokines are also necessary to activate and expand pathogen-specific effector CD8^+ ^T lymphocytes [39]. Therefore, future studies using this neonatal model will focus on how this reduced inflammation may impact the development of a protective memory response in neonates.

## Methods

### Mice and viral infection

Wild type C57BL/6 mice were obtained from Jackson Laboratories. All mice were housed in pathogen-free conditions in a biohazard barrier facility in micro-isolator cages, and all procedures were reviewed and approved by the Saint Louis University Animal Studies Committee. Adult mice were anesthetized, and then inoculated intranasally with 500 pfu/g body weight of SeV, strain 52 (American Type Culture Collection) or with UV-inactivated SeV (UV-SeV) in 30 μl sterile PBS. Two-day old neonatal mice were inoculated intranasally with 500 pfu/g body weight in 6 μl sterile PBS. After infection, mice underwent daily inspection and body weight measurement. To determine lung function, oxygen saturation was measured with the MouseOx™ Pulse-oximeter according to manufacturer's directions (Starr Life Sciences, Oakmont, PA). Three minute readings were taken, readings with error codes were eliminated, and the averages were calculated [40].

### Bronchoalveolar lavage

Bronchoalveolar lavage was performed via tracheal cannulation with PBS containing 2% FBS. A volume of 1.0 ml was used for the adults and 0.3 ml was used for the neonates. Total cell counts were determined after hypotonic lysis to remove erythrocytes. To determine differential cell counts, cells were subjected to cytopsin centrifugation and Wright-Giemsa staining.

### Viral plaque assay

Lungs were weighed and homogenized in 1.0 ml sterile 1X PBS. Serial dilutions of lung homogenates were adsorbed to LLC-MK2 cells for 90 minutes at 37°C with rocking every 15 minutes. After adsorption, cells were washed three times with 1X PBS and a 1% agarose overlay (Molecular Biology Grade, Thermo scientific) was applied. The overlay contained Eagle's Minimum Essential Medium (Lonza, Walkersville, MD), and 0.2 mg/ml trypsin/EDTA (Sigma Aldrich, St. Louis, MO). After 5 days, the cells were fixed with 10% neutral buffered formalin and viral plaques were counted after crystal violet staining. The number of viral plaques per ml were normalized to total grams of lung tissue.

### RNA analysis

Total RNA was isolated from lungs using the Illustra RNAspin Mini RNA extraction kit (GE Healthcare, Buckinghamshire, UK). The High Capacity Reverse Transcription kit (Applied Biosystems, Carlsbad, CA) was used to generate cDNA. Quantitative real-time PCR was performed using the MJ Chromo 4 detection system (Bio-Rad Laboratories, Hercules, CA) using SYBR Green Master Mix (Sigma Aldrich, St. Louis, MO). The specificity of amplification was assessed for each sample by dissociation curve analysis, and the size of the amplicon was confirmed by agarose gel electrophoresis. Expression of each gene was normalized to GAPDH levels and assessed using the 2(-Delta Delta C(T)) method [41]. The following primers were used to determine the expression of the target genes: ICAM-1: forward, 5'- CGC TGT GCT TTG AGA ACT GT -3'; reverse, 5'- GGT GAG GTG CTT GCC TAC TT-3'; IFN-β: forward, 5'- TCC AGC TCC AAG AAA GGA CGA ACA -3', reverse, 5' - TCT GGA GCA TCT CTT GGA TGG CAA - 3'; GAPDH: forward, 5'- AGA TTG TCA GCA ATG CAT CC -3'; reverse, 5'- ACA GTC TTC TGG GTG GCA GT-3'; LGP2: forward, 5'- AGC TGC TGA TGC ATA ACC CAA AGC -3'; reverse, 5'- TGC TGC TCA TAC ATC TGG GTT CCA - 3'; MDA-5: forward, 5'- CGA TCC GAA TGA TTG ATG CA - 3'; reverse, 5'- AGT TGG TCA TTG CAA CTG CT - 3'; CXCL2: forward, 5' - AAA GTT TGC CTT GAC CCT GAA GCC - 3'; reverse, 5'- TCC AGG TCA GTT AGC CTT GCC TTT- 3'; RIG-I: forward, 5'- AGA GAA TTC GGC ACC CAG AA - 3'; reverse, 5'- AGC TCT CGC TCG GTC TCA TC - 3'; SeV: forward, 5' - ATA CGG CAA CCC AGA TGA AG - 3'; reverse, 5'-TCG AAC AAG CTT AGC CCA GT - 3'; TLR-3: forward, 5'- GTG AGA TAC AAC GTA GCT GAC TG - 3'; reverse, 5'- TCC TGC ATC CAA GAT AGC AAG T- 3'; TLR-7: forward, 5'- CGA TCC GAA TGA TTG ATG CA - 3'; reverse, 5'- GGT AAG GGT AAG ATT GGT GGT G - 3'; TLR-8: forward, 5' - GAA AAC ATG CCC CCT CAG TCA - 3'; reverse, 5'- CGT CAC AAG GAT AGC TTC TGG AA - 3'.

### Immunohistochemistry

Lungs were fixed in neutral buffered formalin (at 25 cm water pressure), dehydrated in ethanol, embedded in paraffin, and cut into 5 μm sections. Tissue sections were blocked with normal goat serum and then incubated with 1:500 dilution of hamster anti-mouse primary anti-ICAM Ab (3E2 Pharmingen). Primary antibody was detected with biotinylated goat anti-hamster IgG (1:200 dilution for 30 minute at room temperature) in 3% normal goat serum and the VECTASTAIN IN ABC-AP kit. Slides were then stained with alkaline phosphatase red substrate (Vector Laboratories) and counter stained with hematoxylin.

### ELISA

Lungs were homogenized in 1% Nonidet P-40, 0.05 M Tris, pH 8.0, 250 mM NaCl, 1 mM EDTA containing protease inhibitor cocktail (Sigma Aldrich, St. Louis, MO). Total protein concentrations were determined with Pierce BCA protein assay kit (Thermo Scientific, Rockford, IL) and samples were normalized to equivalent protein concentrations. Protein levels for IFN-γ, TNF-α, and IL-6 were measured using Ready-Set-Go colorimetric sandwich ELISA kits (eBiosciences, San Diego, CA. CXCL2 was measured using the Quantikine Mouse CXCL2/MIP2 ELISA kit (R&D Systems, Minneapolis, MN).

### Western blotting

Lungs were homogenized in 1% Nonidet P-40, 0.05 M Tris, pH 8.0, 250 mM NaCL, 1 mM EDTA containing protease inhibitor cocktail (Sigma Aldrich, St Louis, MO), Total protein concentration were determined with Pierce BCA protein assay kit (Thermo Scientific, Rockford, IL). Primary antibodies were rat anti-mouse RIG-I (Biolegend), rabbit anti-mouse Mda-5 (Cell Signaling), and rabbit anti-mouse GAPDH (Cell Signaling). Secondary antibodies were HRP-conjugated goat anti-rat IgG (Biolegend), goat anti-rabbit IgG (Cell Signaling), donkey anti-rabbit IgG (Biolegend), respectively. Bands were then visualized using Immobilon western chemiluminenscent substrate kit (Millipore, Billerica, MA) and imaged using Fujifilm corporation LAS-400 Chemiluminescent imaging unit.

### Statistical analysis

All values represent means ± standard deviation (SD). Means were compared by a paired-samples *t*-test using IBM^® ^SPSS^® ^Statistics Version 19. *P *values < 0.05 were considered statistically significant.

## Abbreviations

BAL: bronchoalveolar lavage; CXCL2: Chemokine (C-X-C motif) ligand 2; ICAM-1: intracellular adhesion molecule-1; IFN: interferon; Lgp2: laboratory of genetics and physiology 2; Mda-5: melanoma differentiation-associated gene 5; pfu: plaque forming unit; PMN: polymorphonuclear cells; PRR: pattern recognition receptor; PVM: pneumonia virus of mice; RIG-I: retinoic acid-inducible gene; RLR: retinoic acid-inducible gene (RIG)-I-like receptor; RSV: respiratory syncytial virus; SeV: Sendai virus; STAT: signal transducer and activator of transcription; TLR: toll-like receptor; TNF: tumor necrosis factor; UV: ultraviolet.

## Competing interests

No competing financial interests exist.

## Authors' contributions

SB performed viral inoculations, pulse oximetry, viral plaque assay, real-time PCR, and western blot analysis. BTB performed ELISA, and real-time PCR. AMJ performed ELISA, real-time PCR and immunostaining. LPS conceived and designed the study, performed data analysis, and prepared the first draft of the manuscript. All authors read and approved the final version of this manuscript.
